# Driving Antibiotic Resistance Evolution of *E. coli* by Three Commonly Used Disinfectants Under Concentration-Increasing Stress

**DOI:** 10.3390/microorganisms14030616

**Published:** 2026-03-10

**Authors:** Tianchen Wang, Yongqi Li, Yanyang Li, Mengqi Chai, Hangfei Bai, Song Jiang, Jun Xia

**Affiliations:** 1 College of Animal Sciences, Shihezi University, Shihezi 832000, China; 18083933115@163.com (T.W.); 18865345100@163.com (Y.L.); cmqtgzy@163.com (M.C.); xiaobailajia123@163.com (H.B.); 2 College of Animal Science and Technology, Southwest University, Chongqing 402460, China; 14719869471@163.com; 3 Institute of Veterinary Medicine, Xinjiang Academy of Animal Sciences, Urumqi 830011, China

**Keywords:** disinfectants, *Escherichia coli*, subinhibitory concentration, resistance induction, antimicrobial resistance

## Abstract

Antimicrobial resistance (AMR) has become a major global public health challenge, and widely residual disinfectants in the environment are one of the key drivers of bacterial AMR development. This study aimed to investigate the inductive effects of three commonly used disinfectants—benzalkonium bromide (BAB), glutaraldehyde (GTA), and povidone-iodine (PVP-I)—on the resistance of *Escherichia coli* (*E. coli*), as well as the resultant bacterial phenotypic and genetic alterations. Three disinfectants frequently detected in clinical and environmental settings were selected as the research objects: first, their bactericidal efficacy against environmental bacteria was determined; subsequently, a concentration-increasing gradient approach was adopted to systematically explore the evolutionary patterns of *E. coli* resistance under the stress of sub-inhibitory concentrations (SICs). After induction, the bacterial resistance levels to disinfectants and various antibiotics, growth characteristics, and biofilm-forming ability were detected, and combined with whole-genome analysis to investigate genetic-level changes. The results showed that all three disinfectants could enhance *E. coli* resistance to themselves (12–48-fold) and antibiotics, and the induced antibiotic resistance exhibited favorable genetic stability. Among them, BAB induced the strongest resistance, with the most significant increase in resistance levels to multiple antibiotics (16–64-fold); GTA had the weakest inductive effect, only slightly enhancing bacterial resistance to a small number of antibiotics. Notably, all induced strains exhibited reduced growth rates yet markedly enhanced biofilm-forming capacity, alongside acquired genomic structural variations. Their gene functions displayed shared adaptive signatures in coping with environmental stress, while core pathogenicity-associated genes remained conserved. This study demonstrates that inducing *E. coli* using environmentally relevant low concentrations of disinfectant residues as initial induction doses drives the evolution of bacterial antimicrobial resistance (AMR), with distinct resistance induction risks among the three disinfectant types. These findings offer critical insights for standardizing disinfectant application, mitigating the transmission of bacterial AMR, and underscore the imperative of interdisciplinary collaboration to tackle the environmental risks posed by disinfectant residues.

## 1. Introduction

AMR has emerged as one of the most severe global public health challenges of the 21st century. Its core conundrum lies in the fact that the evolutionary rate of drug-resistant bacteria far outpaces the research and development progress of novel antimicrobial agents. This disequilibrium not only directly impairs the efficacy of clinical infection treatment but also poses multifaceted and persistent threats to human health security, food safety supply and the balance of ecological systems [1]. The 2023 Combatting Superbugs report by the UNEP has further quantified this risk: without targeted interventions in the future, AMR could lead to an additional 10 million annual deaths worldwide by 2050, and the global gross domestic product is projected to shrink by US$34 trillion each year due to cascading effects such as surging healthcare costs and declining agricultural productivity [2]. These figures clearly indicate that AMR prevention and control is no longer a challenge confined to the single field of medicine, but a global issue that requires interdisciplinary collaboration across the environmental, medical and agricultural sectors to address.

For a long time, the academic community has generally regarded the irrational use of antibiotics (e.g., clinical overuse [3], agricultural growth-promoting supplementation [4]) as the core driver of the emergence and spread of bacterial resistance. However, interdisciplinary studies in environmental microbiology and public health in recent years have gradually revealed that, in addition to antibiotics, chemical disinfectants in the environment also act as a key non-antibiotic selective pressure [5], and this pressure has intensified exponentially in the wake of the global COVID-19 pandemic [6]. In the post-pandemic period, disinfectants have completely transitioned from common tools for specific settings such as medical care and food processing to routine supplies for public health, with their usage volume rising markedly compared with the pre-pandemic level [7]. Yet this expansion of application scenarios has not been accompanied by the simultaneous popularization of proper disinfection knowledge: most users only focus on disinfection frequency, while lacking sufficient awareness of such key steps as dosage control, scenario applicability matching and waste liquid disposal [8]. Such mismatches in these aspects render the three most widely used disinfectants—BAB, GTA and PVP-I—highly susceptible to amplified risks of environmental residues due to improper operation in daily use. The massive usage volume coupled with non-standard operational practices ultimately allows these three types of disinfectants to enter natural water bodies and soil via medical wastewater, domestic sewage, industrial effluent and other pathways, leading to persistent residues on a pan-environmental scale [9].

Reported residual concentrations of various disinfectants in sewage treatment plants, hospital wastewater and other contaminated environments generally range from 0.01 to 30 mg/L [10,11,12,13], which are far lower than the minimum inhibitory concentrations of these three disinfectants against common environmental bacteria, including *E. coli* [11,14], thus falling into the typical range of sub-inhibitory concentrations (SICs). Such low-concentration, widespread exposure does not directly kill bacteria, but exerts cryptic selective pressure on microorganisms through multiple mechanisms: for instance, inducing the mutation and expression of antibiotic resistance genes, facilitating the horizontal transfer of resistance plasmids, activating efflux pump systems, or triggering microbial adaptive responses by interfering with cellular metabolism, disrupting cell membrane integrity and inducing oxidative stress [15]. Existing studies have provided empirical evidence for this phenomenon: exposure of *Pseudomonas aeruginosa* to SICs of chlorine-containing disinfectants can enhance its resistance to tetracycline, carbenicillin and sulfamethoxazole-trimethoprim [16]; low-concentration exposure to chlorophenols, benzalkonium chloride, glutaraldehyde, chlorhexidine and povidone-iodine can reduce the susceptibility of *E. coli* to subsequent antimicrobial agents by enhancing its biofilm-forming ability [17].

However, existing studies still have three notable limitations, which make it difficult to underpin the accurate assessment and prevention and control of the environmental risks posed by disinfectants. First, there is a lack of systematic comparison of the resistance-inducing effects of BAB, GTA and PVP-I—the three disinfectants frequently detected in both clinical and environmental settings—which has failed to clarify the differences in risk levels among disinfectants of different chemical types. Second, most studies adopt a single SIC design, ignoring the actual environmental scenarios where disinfectant residues form a gradient of low → moderate → high concentrations (still below the MIC) through cumulative effects, which may result in a disconnect between research conclusions and practical applications. Third, in-depth investigations have not been conducted on the fitness costs of environmental adaptation and stability under stress-free conditions of drug-resistant strains induced by disinfectants, as well as the regular patterns of phenotypic and genotypic changes during the process of resistance evolution.

*E. coli*, a common member of the human intestinal microbiota and a model strain for environmental microorganisms, is not only an important vector for the dissemination of resistance genes [18], but, also, its resistance evolution patterns provide crucial reference value for assessing the environmental risks of antimicrobials. In view of this, to address the knowledge gap regarding the inductive risks posed by aldehyde-based, iodine-containing and quaternary ammonium salt disinfectants to *E. coli*, this study selects BAB, GTA and PVP-I—three disinfectants frequently detected in both clinical and environmental settings—and establishes increasing concentration gradients within a strictly defined range of environmentally residual concentrations to systematically investigate the inductive effects of SIC disinfectants on the development of resistance in standard *E. coli* strains. This study aims to clarify the following four key aspects: (1) whether increasing exposure to SICs of the three disinfectants can drive the emergence and enhancement of resistance in *E. coli*; (2) whether there are differences in the inductive effects of different types of disinfectants and their underlying mechanisms; (3) the environmental fitness costs of evolved clones and the stability of induced resistant *E. coli* strains under stress-free conditions; (4) the regular patterns of phenotypic and genotypic changes acquired by the bacteria through adaptive evolution under simulated environmental stress from sub-inhibitory concentrations of disinfectants.

## 2. Materials and Methods

### 2.1. Bacterial Strain, Chemicals and Culture Media

*E. coli* ATCC 25922 was purchased from Shanghai Luwei Biotechnology Co., Ltd., Shanghai, China; Yeast Extract, sodium chloride and Tryptone were purchased from Wuhan Servicebio Technology Co., Ltd., Wuhan, Hubei Province, China; Eosin Methylene Blue agar (EMB) and Brain Heart Infusion agar (BHI) were products of Qingdao Haibo Biotechnology Co., Ltd., Qingdao, Shandong Province, China.

GTA (specification: 5%), PVP-I were purchased from Zhenjiang Zhonglian Guomu Animal Husbandry Technology Co., Ltd., Zhenjiang, Jiangsu Province, China; BAB was purchased from Shandong Mingde Medical Technology Co., Ltd., Dezhou, Shandong Province, China. Doxycycline hydrochloride (DOXY) was obtained from Ruicheng Xianweifu Veterinary Pharmaceutical Co., Ltd., Ruicheng, Shanxi Province, China; gentamicin sulfate injection (GM) from Hebei Rongjiarun Biotechnology Co., Ltd., Handan City, Hebei Province, China; florfenicol injection (FFC) from Zhengzhou Ruiteng Bioengineering Co., Ltd., Zhengzhou, Henan Province, China; oxytetracycline injection (OXY) from Ruicheng Lvman Biopharmaceutical Co., Ltd., Ruicheng, Shanxi Province, China; tetracycline hydrochloride (TC) for injection from Hebei Chengshengtang Veterinary Pharmaceutical Co., Ltd., Shijiazhuang City, Hebei Province, China; enrofloxacin injection (ENR) from Hongbao Veterinary Pharmaceutical Co., Ltd., Yuncheng City, Shanxi Province, China; and ceftiofur sodium for injection (PG) from the corresponding commercial supplier, Sichuan Jishan Zhijia Pharmaceutical Co., Ltd., Chengdu City, Sichuan Province, China.

### 2.2. Bactericidal Activity of Disinfectants Against E. coli

*E. coli* isolated and preserved from the environment was taken out and cultured overnight. On the next day, the bacterial culture was streaked onto EMB and incubated for 16–18 h. After incubation, colonies were observed, and those displaying a metallic luster were confirmed and stored at 4 °C for later use.

In accordance with the neutralizer test results [12] and the quantitative bactericidal assay for bacterial suspensions established in our previous study (the detailed protocol is provided in Appendix A.1), the bactericidal rates of 5% GTA, BAB, and PVP-I against *E. coli* isolated from the environment were determined, so as to evaluate the effectiveness of the disinfectants in the subsequent study.

### 2.3. Determination of MIC Values of the E. coli ATCC25922 Strain

The *E. coli* ATCC 25922 strain was inoculated into 30 mL of LB broth and cultured in a shaker at 37 °C and 190 rpm for 12–16 h until a cell density of 10^8^ CFU/mL was reached. The bacterial suspension was then stored at 4 °C for further use. Taking GTA as an example, the macrodilution broth method was adopted for the assay. Fifteen 10 mL test tubes were prepared, numbered 1 to 15, and each tube was filled with 4 mL of sterilized BHI broth. Four mL of GTA was added to Tube 1 and mixed thoroughly; subsequently, 4 mL of the mixture was transferred to Tube 2, and this serial dilution step was repeated sequentially up to Tube 13. Forty μL of diluted bacterial suspension to be tested was inoculated into Tubes 1 to 14, with no bacterial inoculation performed in Tube 15. Tube 14 served as the disinfectant-free control group and Tube 15 as the blank control group. All test tubes were incubated in a shaker at 37 °C and 190 rpm for 18–24 h prior to result observation. The 0.5MIC of GTA was set as the initial induction concentration, and the identical procedure was applied for the determination of the MIC of BAB and PVP-I. Three replicates were conducted for each disinfectant, and the MIC was defined as the lowest concentration at which no bacterial growth was observed.

### 2.4. Resistance Induction Assay of the E. coli ATCC25922 Strain

Taking GTA as an example, BHI broth was used as the basal medium for the assay, which was autoclaved for 15 min and then cooled to room temperature for subsequent use. The bacterial suspension was prepared by picking single colonies of the target strain from EMB agar plates, inoculating them into 5 mL of sterile BHI broth, and culturing the mixture with constant temperature shaking at 37 °C and 190 rpm to the logarithmic growth phase. The GTA solution was filtered through a 0.22 μm sterile filter membrane for sterilization prior to use to eliminate interference from microbial contamination. The minimum inhibitory concentration (MIC) of GTA against the target strain was determined via the macrodilution broth method, and all subsequent induction concentrations were set based on this MIC value.

An initial 20 mL induction system was established using 50 mL sterile centrifuge tubes. Each tube was added to with BHI broth, 0.2 mL of the bacterial suspension in logarithmic growth phase (at a volume ratio of 1:100 to the total volume), and the calculated volume of GTA stock solution derived from the dilution formula; the additives were pipetted using a sterile micropipette (Eppendorf SE, Hamburg, Germany) and the tube was inverted gently to mix uniformly, ensuring homogeneous distribution of the disinfectant. The above centrifuge tubes were incubated in a constant temperature shaking incubator at 37 °C and 190 rpm. The OD_600_ values were measured using an ultraviolet-visible spectrophotometer (Shanghai Yidian Analytical Instruments Co., Ltd., Shanghai, China) every 2 h, with sterile LB broth containing 0.5 × MIC GTA serving as the blank control. Incubation was terminated when the OD_600_ reached 1.0 ± 0.05. Subsequently, the bacterial suspension was rapidly mixed with 50% sterile glycerol at a volume ratio of 1:1, and the mixture was aliquoted into sterile cryopreservation tubes (1.5 mL per tube). The tubes were labeled with the strain number, induction concentration, passage number and storage date, and immediately stored in a −80 °C refrigerator. At least 3 backup tubes were prepared for each passage to ensure experimental continuity.

Gradual resistance induction was initiated at an initial concentration of 0.5 × MIC, followed by a 2-fold gradient increase (0.5 × MIC → 1 × MIC → 2 × MIC → 4 × MIC…). The gradient setting was based on the rate of bacterial resistance enhancement observed in the pre-experiment to ensure a moderate induction pressure. At least 3 consecutive stable passages were required at each induction concentration, and the full procedures of medium preparation, disinfectant addition, inoculation and culture, OD_600_ monitoring, and glycerol preservation were repeated for each passage. A passage was defined as stable only if 2 criteria were met: the OD_600_ reached 1.0 ± 0.05 within 16–24 h of culture, and the time fluctuation for the resuscitated cryopreserved strain to reach an OD_600_ of 1.0 was ≤2 h. Only after confirming stable passage could the strain be inoculated into the medium with the next-highest induction concentration.

After the strain completed 3 stable passages at the current concentration, it was inoculated into the medium with the next-highest concentration. If the OD_600_ reached ≥0.8 after 16–24 h, the strain was judged to be tolerant to the higher concentration and continued to complete 3 stable passages at this new concentration. If the OD_600_ was <0.8, the strain was judged to be intolerant: the strain from the previous stable concentration was then inoculated into sterile LB broth without disinfectant for 1 passage of resuscitation culture (until OD_600_ = 1.0 ± 0.05), and subsequently re-inoculated into the medium with the original induction concentration for repeated induction. This step was performed to prevent strain growth arrest or death caused by excessive disinfectant pressure.

The induction was terminated when the strain completed 15 consecutive passages at a specific maximum tolerable concentration (Cmax) and failed to reach an OD_600_ ≥ 0.8 upon each attempt of inoculation into the medium with the next concentration (Cmax × 2). At this point, the Cmax was defined as the maximum tolerable concentration (MTC) of the strain to GTA. Subsequently, the last passage strain cultured at Cmax was separately inoculated into BHI medium containing Cmax concentration of GTA (disinfectant pressure group) and sterile BHI medium without any disinfectant (disinfectant-free pressure group). Both groups were subjected to 5 consecutive passages, and the induced strains of each passage were preserved at −80 °C using the glycerol preservation method to ensure the continuity and stability of subsequent experiments.

The above entire experimental procedures were strictly replicated for the other 2 disinfectants (BAB and PVP-I). The final induced strains of the BAB group, GTA group and PVP-I group were designated as A1, B1 and C1, respectively.

### 2.5. Phenotypic Determination of Induced Strains

The minimum inhibitory concentrations (MICs) of antibiotics against the strains from the 3 disinfectant pressure groups and the disinfectant-free pressure group were determined via the microbroth dilution method specified by the Clinical and Laboratory Standards Institute (CLSI), with the final antibiotic concentrations set at 128, 64, 32, 16, 8, 4, 2, 1, 0.5 and 0.25 mg/L; triplicate samples were prepared for each group. Meanwhile, two culture systems were established, one with disinfectant pressure (Cmax) and the other without disinfectant pressure. The above strains were cultured in these systems with constant temperature shaking at 37 °C and 190 rpm, and the bacterial biomass (OD_600_) was measured every 2 h for 24 consecutive hours. The crystal violet staining method was used to determine the biofilm-forming capacity (the detailed protocol is provided in Appendix A.2).

### 2.6. Whole-Genome Sequencing and Bioinformatics Analysis

Scanning Electron Microscopy (SEM), synteny analysis and whole-genome DNA sequencing were performed on the 3 induced populations (A1, B1, C1) evolved from disinfectant treatment. The term population here refers to the mixed population of bacterial cells collected en masse from the evolved cultures at the end of the experiment, which represents the intrapopulational genetic diversity of the bacteria in each treatment group following their adaptation to disinfectants (the detailed protocol is provided in Appendix A.3 and Appendix A.4).

## 3. Results

### 3.1. Bactericidal Efficacy of Disinfectants

**Figure 1:**
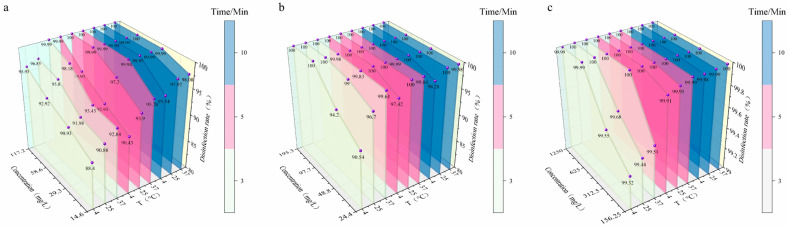
Bactericidal rates of three disinfectants against *E. coli* under different conditions. (**a**): Bactericidal rate of BAB; (**b**): Bactericidal rate of GTA; (**c**): Bactericidal rate of PVP-I. The *X*-axis represents disinfectant concentration, the *Y*-axis represents disinfection efficiency, and the *Z*-axis represents different temperatures. Different colors represent different contact times (3, 5, 10 min).

Based on the bactericidal rate data of PVP-I, BAB and GTA against the target bacteria at different concentrations, temperatures and exposure times, their bactericidal characteristics and disinfection performance were systematically analyzed (Figure 1, Table 1). PVP-I exhibited weak concentration dependence and strong temperature adaptability: at high concentrations (1250 and 625 mg/L), the bactericidal rate reached 100% under all temperature conditions; at the medium concentration (312.5 mg/L), the bactericidal rate was slightly lower (99.55% and 99.68%) after 3 min of exposure at 4 °C and 25 °C, and increased to 100% when the exposure time was extended to 5 min; at the low concentration (156.25 mg/L), the bactericidal rate rose from 99.32% to 99.51% with increasing temperature after 3 min of exposure, and reached ≥99.98% under all temperature conditions after 10 min of exposure, demonstrating a distinct time compensation effect.

In contrast, the bactericidal efficacy of BAB was highly sensitive to both concentration and temperature. A 100% bactericidal rate was only achieved at the high concentration (117.2 mg/L) after 5 min of exposure at 37 °C and 25 °C, and after 10 min of exposure at 4 °C. At medium and low concentrations (58.6, 29.3 and 14.6 mg/L), a stable 100% bactericidal rate could not be attained under all temperature conditions, with the efficacy declining significantly at low temperatures and low concentrations; for instance, the bactericidal rate was merely 95.54% at 14.6 mg/L after 10 min of exposure at 4 °C.

GTA, by comparison, exhibited the optimal temperature stability and concentration adaptability. At high concentrations (195.3 and 97.7 mg/L), the bactericidal rate was 100% under all temperature and exposure time conditions. At the medium concentration (48.8 mg/L), the bactericidal rate was only 94.20% after 3 min of exposure at 4 °C, and rose to 100% with the exposure time extended to 5 min. At the low concentration (24.4 mg/L), a 100% bactericidal rate was achieved after 5 min of exposure at 25 °C, and the bactericidal rate still reached 98.28% after 10 min of exposure at 4 °C, with its bactericidal performance at low temperatures and low concentrations far surpassing that of the other two disinfectants.

### 3.2. Results of MIC Determination by Standard Broth Macrodilution Method

The MIC values of benzalkonium bromide (BAB), glutaraldehyde (GTA) and povidone-iodine (PVP-I) are presented in Table 1.

### 3.3. Determination Results of MIC Values of Induced Strains

**Figure 2:**
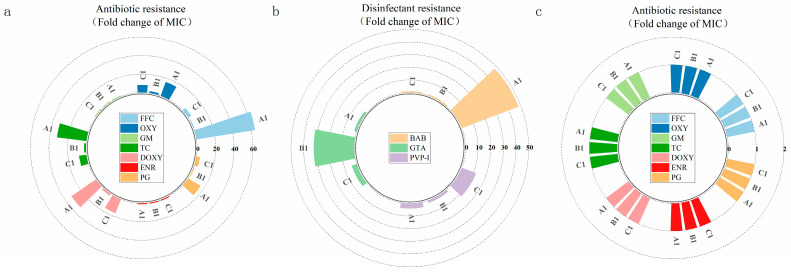
Resistance test of induced strains to disinfectants and antibiotics. (**a**): Determination results of MIC values of bacteria to antibiotics; (**b**): Determination results of MIC values of bacteria to disinfectants; (**c**): Comparison of MIC values of antibiotics for induced strains after five consecutive passages under disinfectant-free conditions and non-passaged final induced strains.

To investigate the effects of long-term disinfectant induction on the resistance evolution of the *E. coli* ATCC 25922 strain, we conducted continuous passage induction experiments with BAB, GTA and PVP-I as inducers, respectively. Ultimately, the BAB group completed 163 passages of induction, the GTA group 145 passages, and the PVP-I group 125 passages. Determination of the disinfectant minimum inhibitory concentration (MIC) values of the final induced strains revealed a significant increase in the resistance of each strain to its inducing disinfectant (Figure 2b), with divergent changes in resistance to non-inducing disinfectants: the MIC value of A1 to BAB was 48-fold higher than that of the original strain, while no significant changes were observed in its MIC values to GTA and PVP-I; the MIC value of B1 to GTA was elevated 32-fold, and its MIC values to BAB and PVP-I increased by 2-fold and 4-fold, respectively; the MIC value of C1 to PVP-I rose 12-fold, with its MIC values to BAB and GTA increasing by 4-fold and 2-fold, respectively (Figure 2a).

In the analysis of antimicrobial resistance, distinct resistance divergence was observed among the different final induced strains (Figure 1a). For strain A1, among the seven tested antimicrobials (gentamicin, enrofloxacin, florfenicol, ceftiofur sodium, doxycycline hydrochloride, tetracycline hydrochloride, oxytetracycline), the MIC values showed extremely significant increases (16–64-fold) for all except gentamicin and enrofloxacin—whose MIC values were consistent with those of the original strain—with the most remarkable enhancement in resistance to florfenicol (64-fold). The changes in the antimicrobial resistance of strain B1 were negligible, with only a 2-fold increase in the MIC values to doxycycline hydrochloride and tetracycline hydrochloride, and no alterations in resistance to the other five antimicrobials. Strain C1 exhibited significant resistance increases (4–16-fold) to five antimicrobials excluding gentamicin and enrofloxacin. To verify the stability of the induced resistance, each final induced strain was subjected to five consecutive passages in disinfectant-free medium. The results showed no significant fluctuations in their MIC values to antimicrobials (Figure 2c), indicating that the disinfectant-induced antimicrobial resistance possessed favorable genetic stability.

### 3.4. Phenotypic Determination Results of Induced Strains

**Figure 3:**
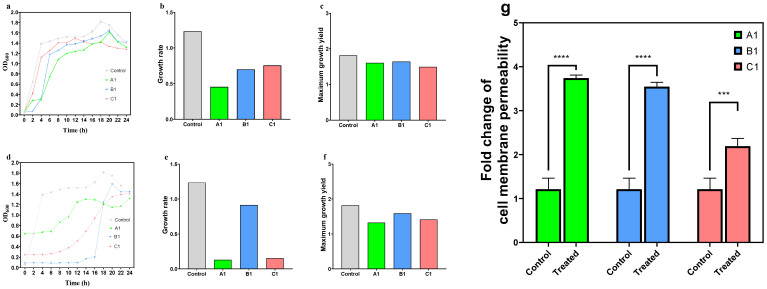
Phenotypic determination results of induced strains. (**a**): Bacterial growth curves under disinfectant-free conditions; (**b**): Bacterial growth rates under disinfectant-free conditions; (**c**): Final bacterial biomass yield under disinfectant-free conditions; (**d**): Bacterial growth curves under disinfectant pressure; (**e**): Bacterial growth rates under disinfectant pressure; (**f**): Final bacterial biomass yield under disinfectant pressure; (**g**): Bacterial biofilm-forming ability. *** = *p* < 0.001, **** = *p* < 0.0001.

Under disinfectant-free conditions, the original control strain (Control) exhibited robust growth kinetic characteristics, rapidly entering the logarithmic growth phase and reaching a high plateau phase. Its growth rate and maximum biomass yield were significantly higher than those of the disinfectant-induced strains (A1, B1, C1), thus indicating that the disinfectant induction process imposed a fitness cost on bacterial growth, with the magnitude of this cost varying by disinfectant type (Figure 3). Among the induced strains, A1 (BAB-induced) showed the most pronounced growth inhibition, with a growth rate of only 36.8% and a maximum biomass yield reduced to 88.5% of the control strain. In comparison, the growth inhibition of B1 (GTA-induced) and C1 (PVP-I-induced) was relatively mild: the growth rate and maximum biomass yield of B1 were 56.7% and 90.5% of the control strain, respectively, while those of C1 were 61.1% and 82.2%, respectively. These findings reflect distinct specific differences in the impacts of different disinfectant inductions on bacterial growth functions.

Under the corresponding disinfectant pressure conditions, each induced strain displayed a divergent growth adaptation pattern (Figure 3). A1 exhibited a “slow adaptation” characteristic: early-stage growth was almost arrested, followed by a gradual entry into the logarithmic growth phase and a stable plateau phase at the later stage, with an ultimately low growth rate and a maximum biomass yield of 1.323. B1 showed a “delayed burst” growth dynamic: growth exhibited almost no obvious change in the first 14 h, after which the growth rate increased sharply at 18 h and rapidly reached the maximum biomass yield; this growth profile suggested that the strain may achieve delayed tolerance to GTA through mechanistic activation. C1 presented a “sustained gradual” growth mode, with OD values increasing progressively with incubation time, which reflected a stepwise adaptation process to PVP-I pressure.

The results of the crystal violet staining assay demonstrated that, compared with the original control strain, all disinfectant-induced strains exhibited a significant enhancement in biofilm-forming ability (*p* < 0.001), with a distinct hierarchical difference in the magnitude of this enhancement (Figure 3). A1 (BAB-induced) and B1 (GTA-induced) showed the most remarkable increases in biofilm-forming ability, reaching 3.08-fold and 2.83-fold that of the original strain, respectively (both *p* < 0.0001). Although the enhancement magnitude of C1 (PVP-I-induced) was relatively lower, it still reached 1.77-fold that of the original strain (*p* < 0.001).

### 3.5. Bacterial Morphological Changes

**Figure 4:**
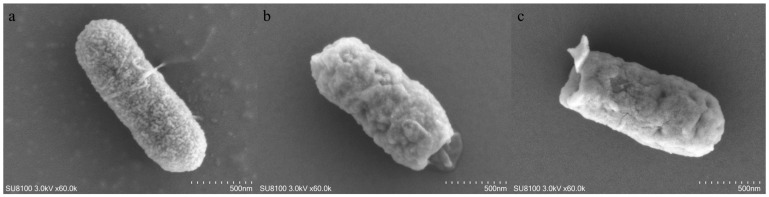
Scanning Electron Microscopy (SEM) image of A1 (**a**), B1 (**b**), C1 (**c**).

A1 (Figure 4a) still retained its rod-shaped structure, yet the surface roughness of the bacterial cells was significantly increased, with densely distributed fine granular protrusions observable; mild linear folds also appeared on the cell surface. In addition, residual flagellar structures were detected at one end of the bacterial cells, and no severe morphological distortion was observed in the overall cell shape.

B1 (Figure 4b) exhibited the most pronounced morphological distortion: the cell surface presented an irregular shrinkage pattern with deepened and disordered folds, and the overall outline lost the regularity of the original strain, with only an obscure short rod-shaped outline identifiable.

C1 (Figure 4c) partially retained its rod-shaped structure, but extensive folds and local protrusions emerged on the cell surface; distinct structural damage (or protrusion) was also visible at one end of the bacterial cells. The degree of overall morphological irregularity was significantly higher than that of A1 but lower than the shrinkage and distortion observed in B1.

### 3.6. Synteny Analysis of Induced Strains and the E. coli ATCC25922 Strain

To elucidate the genomic divergence characteristics between the disinfectant-induced lineages and the reference genome *E. coli* ATCC 25922, we performed whole-genome alignment and fine-scale segmental alignment separately for the three induced lineages.

**Figure 5:**
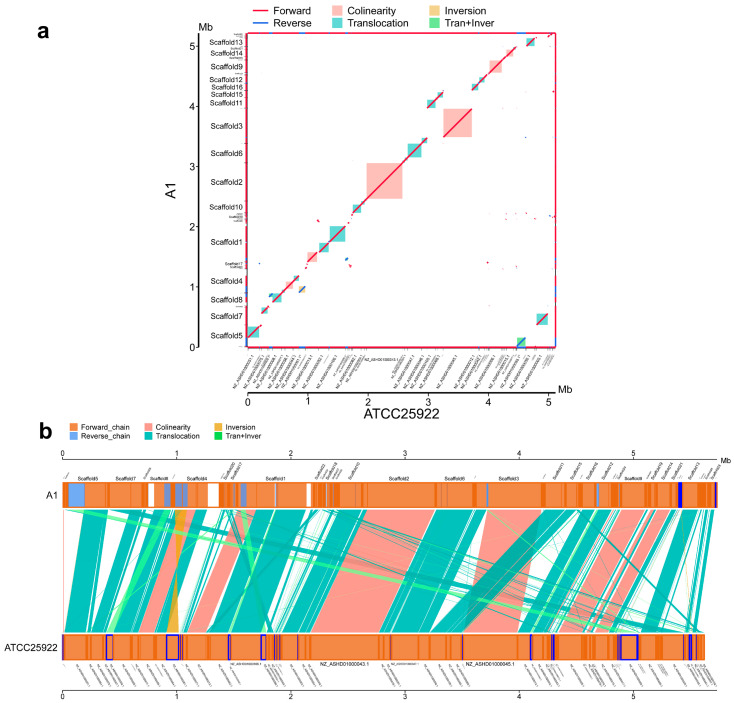
Synteny analysis of induced strain A1 and *E. coli* ATCC 25922. (**a**): Wide-range collinearity, (**b**): local positional arrangement.

**Figure 6:**
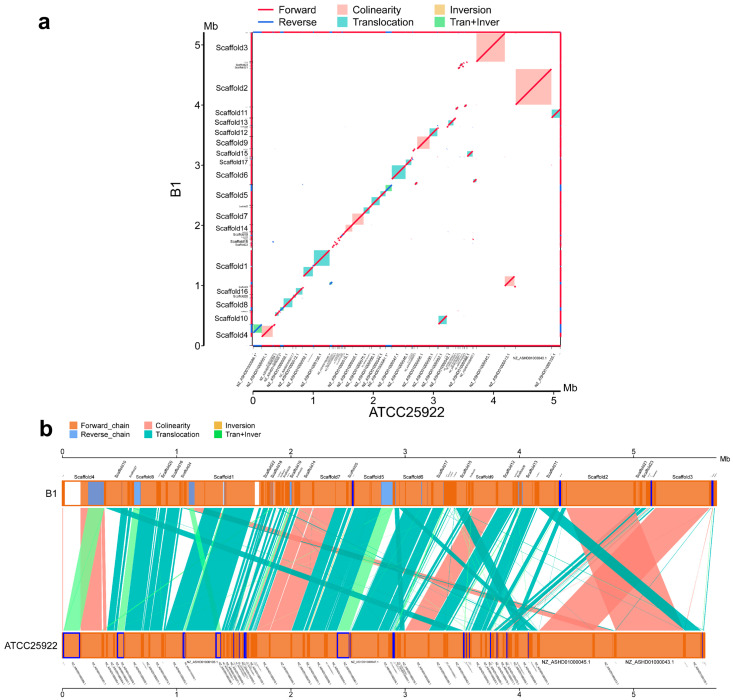
Synteny analysis of induced strain B1 and *E. coli* ATCC 25922. (**a**): Wide-range collinearity, (**b**): local positional arrangement.

**Figure 7:**
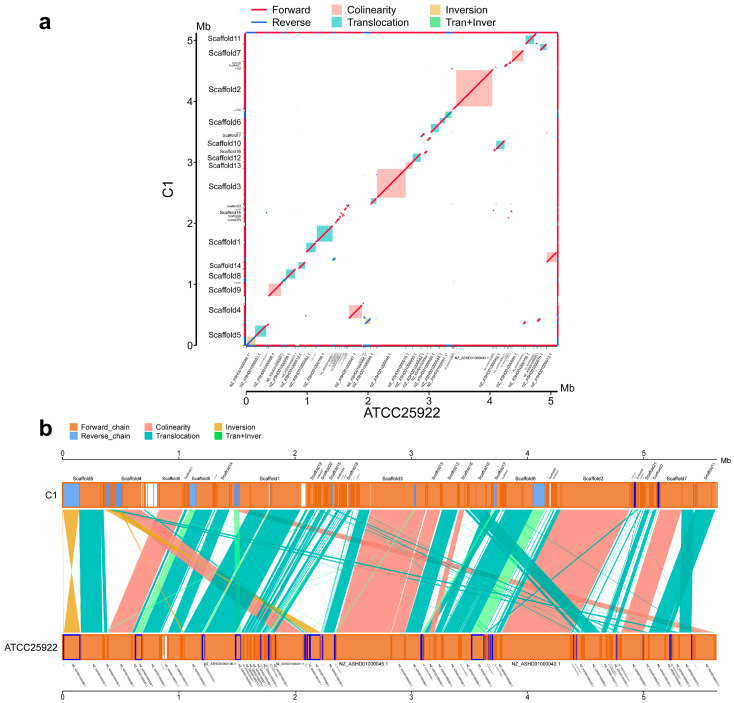
Synteny analysis of induced strain C1 and *E. coli* ATCC 25922. (**a**): Wide-range collinearity, (**b**): local positional arrangement.

All three induced lineages retained syntenic blocks (marked in pink) with *E. coli* ATCC 25922 (a in Figure 5, Figure 6 and Figure 7), which reflects the conservation of ancestral gene linkage relationships. However, the continuity and coverage of synteny, as well as the structural variation profiles, exhibited distinct disinfectant specificity.

For A1 (Figure 5b), the syntenic blocks showed high continuity along the diagonal, with structural variations dominated by translocations and translocation + inversion events. The continuity of syntenic regions in B1 (Figure 6b) was lower than that in A1, and translocations accounted for a higher proportion of its structural variations. The syntenic blocks in C1 (Figure 7b) were the most dispersed, with the coexistence of translocations, inversions and translocation + inversion events, making it the lineage with the most abundant and complex types of variations among the three.

### 3.7. Common Features of Gene Functional Regulation and Core Pathways in E. coli Mutants Induced by Disinfectants

**Figure 8:**
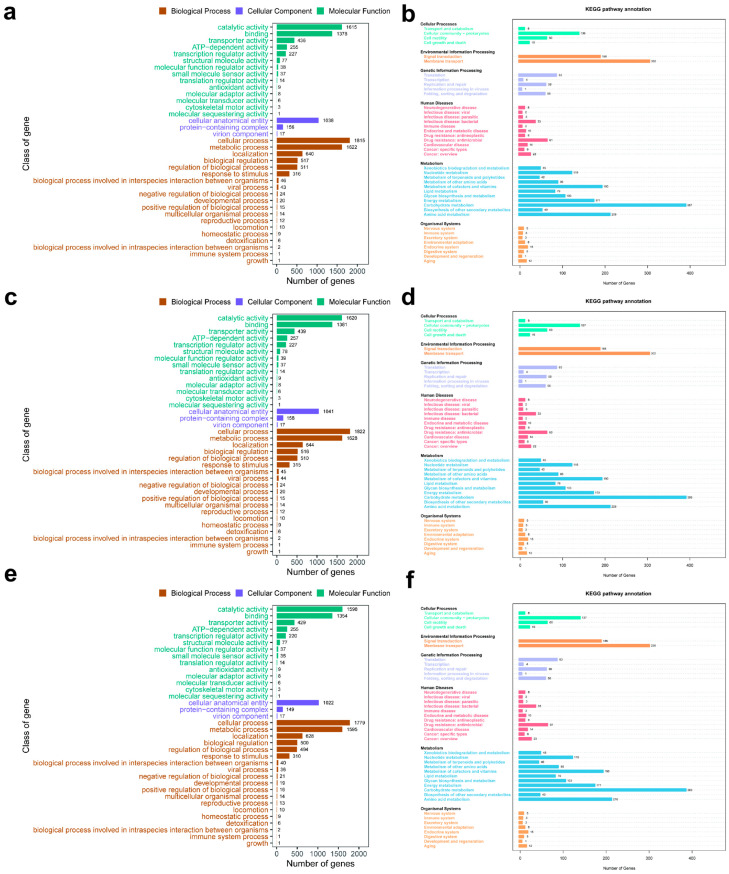
GO and KEGG database annotation. (**a**,**b**): GO and KEGG database annotation of A1; (**c**,**d**): GO and KEGG database annotation of B1; (**e**,**f**): GO and KEGG database annotation of C1.

To elucidate the patterns of gene functional regulation in A1, B1 and C1, we first performed Gene Ontology (GO) enrichment analysis, which covered three major categories: Biological Process (BP), Cellular Component (CC) and Molecular Function (MF). In the MF category (Figure 8a,c,e; bluish-green bands), significant enrichment was observed for binding and catalytic activity in all three groups. This indicates that these molecular functions represent the common foundational features of bacterial responses to disinfectant stress, meaning that the genes associated with molecular binding and catalytic activity were extensively activated at the molecular level in strains induced by any of the three disinfectants to counteract the stress imposed by disinfectants. At the BP level (Figure 8a,c,e; brown bands), cellular process and metabolic process were commonly enriched across the three groups, demonstrating that consistent regulation of gene expression was exhibited in bacterial cellular life activities and metabolite-related biological processes after induction by different disinfectants. Fundamental cellular life activities and metabolic processes thus constitute an important biological basis for bacterial adaptation to disinfectant-exposed environments. In the CC category (Figure 8a,c,e; purple bands), cellular anatomical entity was identified as the core enriched term shared by all three groups. This signifies that the strains displayed common enrichment characteristics in cell structure-related genes upon induction by different disinfectants, and genes associated with the basic structural components of bacterial cells are involved in the response to disinfectant stress.

Furthermore, we conducted Kyoto Encyclopedia of Genes and Genomes (KEGG) pathway enrichment analysis to investigate alterations in bacterial metabolic pathways, signal transduction and disease-related pathways following induction by different disinfectants (Figure 8b,d,f). A total of 2613, 2613 and 2639 protein-coding genes were annotated in the genomes of A1, B1 and C1, respectively; these genes were assigned to 40 pathways within six major functional categories, namely Cellular Processes, Environmental Information Processing, Genetic Information Processing, Human Diseases, Metabolism and Organismal Systems. Although the number of annotated genes varied among the three groups, the distribution trend of annotated genes was consistent across all groups: the largest number of annotated genes were associated with carbohydrate metabolism under the Metabolism category, followed by genes related to membrane transport under Environmental Information Processing and amino acid metabolism under the Metabolism category.

### 3.8. Pathogen–Host Interaction Gene Characteristics and Phenotype Correlations in E. coli Mutants

**Figure 9:**
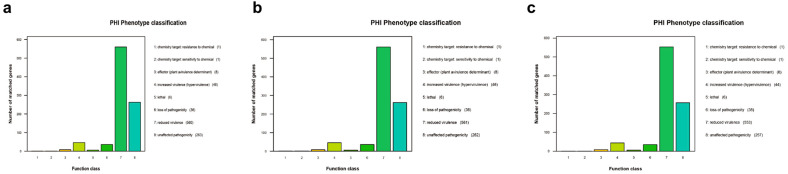
PHI database annotation. (**a**): PHI database annotation of A1; (**b**): PHI database annotation of B1; (**c**): PHI database annotation of C1.

Genomic alignment and annotation of A1, B1 and C1 were performed against the Pathogen Host Interaction database (PHI). The results showed that the number of annotated core PHI-associated genes exhibited a high degree of consistency among the three strains: 921 genes were annotated in both A1 (Figure 9a) and B1 (Figure 9b), while 905 genes were annotated in C1 (a 1.7% reduction compared with the former two, Figure 9c). This indicated that sub-inhibitory concentration induction by different disinfectants (aldehyde-based, quaternary ammonium salt-based and iodine-containing) caused a comparable level of overall perturbation to the core pathogen–host interaction gene pool of *E. coli* ATCC 25922, whereas PVP-I induction might exert a slight effect on the retention or annotation validity of a small number of PHI genes.

Virulence-attenuating genes constituted the most abundant category of PHI genes in all induced strains, with a proportion exceeding 60% in each: 560 such genes were identified in A1 (60.8% of the annotated genes), 561 in B1 (60.9%), and 553 in C1 (61.1%). The three strains were highly consistent in both the number and proportion of virulence-attenuating genes, with only seven to eight fewer such genes in C1 than in A1 and B1.

In contrast, virulence-enhancing genes exhibited strict conservation across the three strains: 46 such genes were present in both A1 and B1 (5.0% of the annotated genes) and 44 in C1 (4.9%), representing a numerical fluctuation of only ±2 genes. The number of pathogenicity-loss genes was also highly consistent among all induced strains, with 36 genes in A1 and B1 (3.9%) and 35 in C1 (3.9%). Lethality-associated genes were completely conserved, with only six genes (0.65%) identified in each of the three strains. These results demonstrated that, during the 125–163 passages of evolutionary induction by disinfectants, *E. coli* lost only a small number of non-core pathogenic genes while maintaining the stability of core lethality-associated genes.

A1, B1 and C1 exhibited an identical mutation profile in terms of pathogenic effector genes, chemical resistance genes and chemical sensitivity genes: eight genes were converted into pathogenic effector genes via mutation (0.87% of the annotated genes) in each strain, one gene acquired chemical resistance through mutation, and one gene developed chemical sensitivity due to mutation in all three strains.

### 3.9. Analysis Results of Virulence Factors (VFDB) in Pathogenic E. coli

This study systematically elucidated the characteristics of virulence factors (VFs) and their associated genes in drug-resistant strains derived from the standard *E. coli* ATCC 25922 induced by sub-inhibitory concentrations of three different disinfectants. Specifically, 68 unique VFs and 326 non-redundant associated genes were annotated in A1, 69 unique VFs and 358 non-redundant associated genes in B1, and 69 unique VFs and 362 non-redundant associated genes in C1. All three strains covered the core processes of pathogenic bacterial virulence, and the functional distribution of their virulence genes was highly coordinated with the disinfectant-induced resistant phenotypes. However, due to differences in the action mechanisms of the disinfectants, there were significant differentiations in the number of genes and their functional preferences.

### 3.10. Analysis Results of Resistance Genes

Integrating whole-genome resistance gene annotation with phenotypic data revealed that the three final strains induced by BAB, GTA and PVP-I all evolved diversified resistance profiles covering 22 categories of resistance genes, with their core resistance mechanisms focusing on the activation of efflux pump systems, modification/inactivation of antibiotic targets, and biofilm regulation.

The core commonality among the three strains lay in the broad-spectrum enrichment and functional conservation of drug-resistant efflux pump genes. The core efflux pump genes including the macB, Mex family (MexB/MexY, etc.), ade family (adeB/adeJ, etc.) and acr family (acrB/acrF, etc.), as well as regulatory factors such as evgS, baeR and rob, were detected in all three strains. The widespread activation of these genes provided a key molecular basis for the significant increase in the MIC of the strains to the inducing disinfectants. Moreover, these genes were co-expressed with the fim family and flg family genes associated with enhanced biofilm-forming ability, which enhanced the tolerance to disinfectants and antibiotics through a dual mechanism of active efflux and physical barrier.

In A1, the β-lactam and chloramphenicol resistance genes exhibited significant enrichment characteristics, and the expression activation levels of core efflux pump genes such as acr and Mex were the highest. This was consistent with the aforementioned phenotypic characteristic of a 16–64-fold increase in resistance to five antibiotics including cephalosporins and florfenicol in this strain. The overall expression activation level of antibiotic resistance genes in B1 (GTA-induced strain) was low, with only the tetracycline resistance genes showing low-level activation. This gene expression characteristic corresponded to the weak resistant phenotype of the strain, which only had a 2-fold increase in MIC to doxycycline and tetracycline. The enrichment and activation levels of resistance genes in C1 (PVP-I-induced strain) were intermediate between those of A1 and B1, with the chloramphenicol, β-lactam and tetracycline resistance genes in a state of moderate activation, corresponding to its 4–16-fold increase in resistance to five antibiotics.

## 4. Discussion

AMR has become a globally urgent public health crisis to address. Beyond the irrational use of antibiotics, chemical disinfectants with widespread environmental residues—acting as a non-antibiotic selective pressure—have attracted growing attention for their pivotal role in driving bacterial resistance [19]. In this study, three disinfectants frequently detected in clinical and environmental settings were selected as the research objects. First, their bactericidal efficacy against environmental bacteria was determined; subsequently, a concentration-increasing gradient design was adopted to systematically investigate the evolutionary patterns of antibiotic resistance in *E. coli* under the stress of SICs. This study revealed the disinfectant type-specific resistance induction effects, genetic stability, and the synergistic adaptation mechanisms of phenotype and genotype in *E. coli*, thus providing critical experimental evidence for the precision prevention and control of AMR transmission driven by disinfectants.

The experimental results demonstrated that the three classes of disinfectants exhibited a significant gradient difference in their resistance induction effects on *E. coli*, forming a risk hierarchy of BAB > PVP-I > GTA, and this disparity was highly coupled with the chemical properties and action mechanisms of the disinfectants. As a quaternary ammonium salt disinfectant, BAB exerts its primary bactericidal effect by disrupting bacterial cell membranes [20]; exposure to its sub-inhibitory concentrations readily imposes strong selection pressure for adaptive mutations such as efflux pump activation and bacterial cell membrane component modification. Whole-genome analysis further confirmed that, in A1, the activation levels of core efflux pump genes including the acr, Mex and ade families, as well as regulatory factors evgS and baeR, were the highest, with significant enrichment of β-lactam and chloramphenicol resistance genes. This molecular signature directly underpinned its strong resistant phenotype, characterized by a 48-fold increase in MIC to the inducing disinfectant and a 16–64-fold elevation in resistance to five antibiotics. In contrast, GTA—an aldehyde-based disinfectant—exerts bactericidal activity via cross-linking with bacterial proteins and features a highly specific action mechanism [21]; the selection pressure under its sub-inhibitory concentrations is milder, which only induces low-level activation of tetracycline resistance genes. This resulted in its induced strain B1 exhibiting a mere 2-fold increase in MIC to doxycycline and tetracycline, with no significant resistance alterations to other antibiotics. PVP-I employs an iodine-releasing bactericidal mechanism that combines membrane damage and oxidative stress effects [22]; the resistance gene enrichment level in its induced strain C1 was intermediate between that of A1 and B1, corresponding to a 4–16-fold increase in resistance to five antibiotics. This clearly reflects the strict coordination between the chemical type of disinfectants and their resistance induction effects.

Notably, none of the three induced strains developed a resistant phenotype to gentamicin (aminoglycoside) and enrofloxacin (fluoroquinolone). Although relevant resistance genes such as kdpE and gyrA were detected in their genomes, no functional activating mutations occurred and their expression was restricted. This characteristic of selective activation indicated that disinfectant selection pressure does not non-specifically activate all bacterial resistance genes, but instead targetedly regulates resistance pathways associated with the disinfectant’s own action mechanism [23]. This finding not only unveils the specific patterns of resistant genotypic-to-phenotypic conversion, but also provides molecular targets for predicting disinfectant-associated cross-resistance risks. Additionally, it fills the knowledge gap in existing research regarding the lack of systematic comparisons among these three frequently detected disinfectants, and lays a direct experimental foundation for the differentiated regulation and management of disinfectants in practical applications.

Building on the clarified gradient differences in resistance induction, further investigations revealed that the antibiotic resistance induced by disinfectants possessed favorable genetic stability. After the final induced strains were subjected to five consecutive passages in disinfectant-free medium, no significant fluctuations were observed in their MIC values to antibiotics. This confirmed that such resistance was not a transient physiological adaptation, but a heritable phenotypic remodeling based on genomic structural variations and resistance gene activation. Whole-genome synteny analysis showed that all three induced strains retained core syntenic blocks with the original strain, yet exhibited disinfectant-specific structural variations (including translocations and inversions). Moreover, the activation of efflux pump genes and biofilm regulatory genes (the fim family and flg family) synergistically acted with the enhanced biofilm-forming ability (1.77–3.08-fold increase), which strengthened the stability of resistance through a dual mechanism of active efflux and physical barrier. This finding also provides critical support for explaining why low-concentration disinfectant residues in the environment can drive the long-term transmission of AMR.

Meanwhile, the acquisition of resistance was not cost-free, but accompanied by a significant fitness cost in environmental adaptation: the growth rates of all induced strains decreased markedly. Specifically, the growth rate of A1 (BAB-induced strain) was only 36.8% of that of the original strain, while those of C1 (PVP-I-induced strain) and B1 (GTA-induced strain) were 61.1% and 56.7% of the original strain, respectively. This growth inhibition was directly associated with the high metabolic cost of resistance gene overexpression, reflecting the evolutionary resource allocation strategy of bacteria under disinfectant stress: prioritizing the maintenance of resistance-associated functions at the expense of partial growth efficiency. Furthermore, under the corresponding disinfectant pressure, the distinct adaptive patterns exhibited by each induced strain—“slow adaptation” in A1, “delayed burst” in B1 and “sustained gradual” adaptation in C1—further embody the specific adaptive mechanisms of bacteria in response to different disinfectant stresses, and also provide a new perspective for understanding the survival strategies of drug-resistant strains in complex environmental settings.

Deeper analyses revealed that disinfectant induction not only drives the evolution of resistance, but also triggers the coordinated remodeling of bacterial phenotypes and genotypes. This coordinated remodeling is first manifested in the intuitive alterations of bacterial cellular morphology. Observations under a Scanning Electron Microscope (SEM, 60.0k×),Hitachi Scientific Instruments (Beijing) Co., Ltd., Beijing, China. showed that each induced strain exhibited distinct morphological distortion: A1 still retained a roughly rod-shaped structure, yet the surface roughness of the bacterial cells was significantly increased, with densely distributed fine granular protrusions and mild linear folds observable, and residual flagellar structures at one end of the cells; B1 displayed the most severe morphological distortion, with the cell surface presenting an irregular shrinkage pattern accompanied by disordered folds and a loose cellular structure; C1 (the PVP-I-induced strain) showed an intermediate level of morphological change between A1 and B1, retaining a partial rod-shaped structure while having extensive folds and local protrusions on the cell surface, as well as distinct structural damage at one cell end.

The degree of morphological distortion exhibited a gradient of B1 > C1 > A1, which is directly linked to the action mechanisms of the disinfectants: the mild morphological alterations of A1 are associated with the cell membrane component modification caused by the activation of its efflux pump genes; the severe shrinkage of B1 corresponds to the cellular structural damage induced by the protein cross-linking effect of GTA; the intermediate morphological state of C1 matches its bactericidal mechanism, which combines membrane damage and oxidative stress effects. Moreover, the differences in the morphological phenotypes are also consistent with the gradient of activation intensity of resistance genes among the various strains.

GO and KEGG enrichment analyses further demonstrated that the three induced strains exhibited shared enrichment characteristics in molecular functions (binding, catalytic activity), biological processes (cellular process, metabolic process) and core pathways (carbohydrate metabolism, membrane transport). This indicates that, regardless of the disinfectant used for induction, bacteria counteract environmental stress by activating genes associated with basic life activities and substance metabolism, thus forming the common foundation for resistance evolution. In contrast, disinfectant-specific phenotypic divergence (e.g., biofilm-forming ability: A1 ≈ B1 > C1) was directly associated with the activation intensity of resistance genes, reflecting a precise genotype–phenotype correspondence.

In addition, PHI database and VFDB analyses further uncovered a clear trade-off between resistance and virulence in the induced strains: the proportion of virulence-attenuating genes exceeded 60% in all induced strains, suggesting that bacteria sacrifice some non-core virulence functions to prioritize the development of resistance. However, core pathogenicity-associated genes (e.g., lethality-associated genes, virulence-enhancing genes) were highly conserved, with only minor fluctuations (e.g., two fewer virulence-enhancing genes in C1 compared with A1). This characteristic indicated that, although the virulence of drug-resistant strains was attenuated, their core infectivity was retained—a finding consistent with the research conclusions of Sébastien B et al. [24]. Thus, the risk of clinical transmission remains non-negligible: even the BAB-induced strain, which displays high resistance to multiple antibiotics, may still cause infections via its core pathogenicity-associated genes. This discovery also emphasizes that AMR prevention and control require cross-scenario coordination between addressing environmental disinfectant residues and managing clinical infections.

Although this study has systematically elucidated the core principles governing *E. coli* resistance evolution driven by the three disinfectants, it still has certain limitations, which in turn point to directions for subsequent research. Firstly, a standard *E. coli* strain (ATCC 25922) was used in this study, whereas strains that naturally harbor resistance genes in the environment may exhibit distinct evolutionary dynamics [25], necessitating further validation with an expanded strain scope. Secondly, although the laboratory-based gradient concentration simulation closely mimics the characteristics of environmental disinfectant residues, it does not account for the synergistic effects of the coexistence of multiple disinfectants or their interactions with other pollutants such as heavy metals and antibiotics [26], thus failing to fully reflect the actual conditions of complex natural environments. Thirdly, this study focuses on short-term evolutionary processes (125–163 passages), and the long-term survival and transmission dynamics of drug-resistant strains in natural environments still require long-term follow-up investigations.

Based on this, future research can be carried out in three aspects. First, incorporate clinically isolated strains and environmental indigenous strains to investigate the strain specificity of disinfectant-induced resistance effects. Second, establish a multi-pollutant synergistic stress model to elucidate the resistance evolution mechanisms under combined environmental stress. Third, combine metagenomic approaches with field monitoring to track the transmission pathways and ecological impacts of drug-resistant strains in aquatic, soil and other environmental matrices. In addition, in-depth exploration of the regulatory networks between resistance genes and virulence genes can lay a theoretical foundation for the development of novel disinfectants or intervention strategies that enable the targeted inhibition of bacterial resistance evolution.

## 5. Conclusions

By simulating the actual cumulative characteristics of disinfectant residues in the environment, this study conducted long-term induction of the standard *E. coli* ATCC 25922 strain using a sub-inhibitory concentration (SICs) increasing gradient protocol, and systematically investigated the resistance-driving effects of three frequently used disinfectants (BAB, GTA and PVP-I). The results confirmed that low-concentration disinfectant residues in the environment can effectively drive *E. coli* to develop resistance with favorable genetic stability, and the induction risks of different types of disinfectants exhibited significant hierarchical differences: BAB posed the highest resistance induction risk, PVP-I an intermediate level, and GTA a relatively low risk. Bacteria adapt to disinfectant stress primarily by activating efflux pump systems, enhancing biofilm formation, and modifying antibiotic targets, while forming distinct evolutionary trade-offs between the acquisition of resistance and growth efficiency (a universal decline in the growth rate of induced strains), as well as virulence functions (attenuation of non-core virulence genes alongside conservation of core pathogenicity-associated genes).

This study achieved the quantitative resistance risk grading of the three frequently used disinfectants under a unified experimental system. It not only filled the research gap caused by the disconnection between traditional single-concentration experiments and the actual cumulative effects of disinfectants in the natural environment, but also provided critical experimental evidence and theoretical support for the accurate risk assessment of environmental disinfectants and the formulation of differentiated prevention and control strategies. The findings not only offer scientific guidance for standardizing the use of disinfectants across all scenarios, but also highlight the necessity of interdisciplinary coordination among the environmental, medical and agricultural fields to address the risks of environmental disinfectant residues. Furthermore, they provide an important practical reference for mitigating the global antimicrobial resistance (AMR) crisis and slowing down the global spread of drug-resistant bacteria.

## Figures and Tables

**Table 1:** MIC of ATCC25922 Against Three Disinfectants.

Pathogenic Microorganism	Disinfectant (mg/L)		
	BAB	GTA	PVP-I (Available Iodine Concentration)
**ATCC25922**	14.6	48.8	15.6

## Data Availability

The original contributions presented in this study are included in the article. Further inquiries can be directed to the corresponding authors.

## Abbreviations

The following abbreviations are used in this manuscript:

BAB	benzalkonium bromide
GTA	dilute glutaraldehyde
PVP-I	povidone-iodine
AMR	antimicrobial resistance
DOXY	doxycycline hydrochloride
GM	gentamicin sulfate injection
FFC	florfenicol injection
OXY	oxytetracycline injection
TC	tetracycline hydrochloride
ENR	enrofloxacin injection
PG	ceftiofur sodium for injection

## Appendix A

### Appendix A.1. Quantitative Bactericidal Test on Bacterial Suspension

Prepare 5 disinfectants according to the instructions, each with 4 concentration gradients (MBC, MIC, 1/2MIC, and 1/4MIC) for different bacterial strains. Prepare 50 mL of each gradient and incubate in a 20 ± 1 °C water bath to achieve equilibrium before use. Concurrently, the concentration of the bacterial suspension was adjusted to 1 × 10^8^–5 × 10^8^ CFU/mL to ensure the accuracy of the test bacterial concentration.

The disinfection reaction system was established as follows [27] (with reference to Laboratory Test Methods for Bactericidal Efficacy of Disinfectants): 0.5 mL of standardized bacterial suspension and 0.5 mL of 6% bovine serum albumin (used as an organic interfering substance) were sequentially added to a sterile test tube. After equilibration in a 37 °C water bath for 5 min, 4 mL of the pre-warmed disinfectant solution (equilibrated to the target temperature) was injected into the tube, followed by vortex oscillation for 30 s to achieve complete mixing.

At preset time points, 0.5 mL of the above mixture was transferred to a test tube containing 4.5 mL of the corresponding neutralizer, and vortex oscillation was conducted for 30 s to terminate the disinfection reaction. Subsequently, 100 μL of the reaction-terminated solution was quantitatively pipetted and inoculated onto nutrient agar plates for microbial quantification. Finally, the inoculated plates were incubated at 37 °C for 24 h, and the number of viable colonies was counted; only plates with ≥30 CFU were considered valid data.

The bacterial killing rate was calculated using the formula: (Viable count in the control group − Viable count in the treatment group)/Viable count in the control group × 100%. Each experiment was independently repeated 3 times, and the final results were expressed as the mean ± standard deviation of the 3 replicates.

### Appendix A.2. Crystal Violet Staining Method

The prepared seed bacterial suspension was inoculated into 96-well cell culture plates at an inoculum size of 1:100, with 200 μL of LB liquid medium added to each well, and the plates were subjected to static incubation in a 37 °C constant temperature incubator for 72 h to facilitate biofilm formation. After biofilm incubation in the 96-well plates, the medium in each well was discarded, and each well was washed once with 1 × PBS buffer to remove unattached planktonic bacteria. Subsequently, 200 μL of 4% methanol fixative was added to each well and incubated at room temperature for 15 min for fixation to stabilize the biofilm structure. After discarding the fixative, each well was gently washed with 300 μL of 1 × PBS buffer to remove residual fixative. 200 μL of 0.1% crystal violet staining solution was then added to each well and stained in the dark at room temperature for 30 min to allow the crystal violet to fully bind to the polysaccharides and proteins in the biofilm extracellular matrix. The staining solution was discarded, and each well was washed 3 times with 1 × PBS buffer to remove unbound free dye. After the plates were air-dried naturally, 200 μL of 75% ethanol solution was added to each well; the plates were gently shaken and incubated at room temperature for 3–5 min to completely dissolve the crystal violet bound to the biofilms. Finally, the absorbance (OD_570_) of each well was measured using a microplate reader (Thermo Fisher Scientific (China) Co., Ltd., Shanghai, China) at a wavelength of 570 nm, and the absorbance value was positively correlated with the relative biomass of the biofilm formed.

### Appendix A.3. SEM Sample Preparation and Imaging

Bacterial cells were collected by centrifugation at 4 °C for SEM observation. The samples were washed 3 times with 0.1 mol/L phosphate buffer (pH 7.2), fixed with 2.5% glutaraldehyde at 4 °C for 24 h, dehydrated with a graded ethanol series, subjected to critical-point drying and gold sputtering, and then observed and imaged at 60.0k× magnification using an SU8100 SEM, Hitachi Scientific Instruments (Beijing) Co., Ltd., BeiJing, China.

### Appendix A.4. Whole-Genome Sequencing and Bioinformatics Analysis

Genomic DNA was extracted using the CTAB method. DNA integrity was verified by 1% agarose gel electrophoresis, and concentration (≥50 ng/μL) and purity (OD260/280 = 1.8–2.0) were determined using a NanoDrop 2000 spectrophotometer, Thermo Fisher Scientific (China) Co., Ltd., Shanghai, China. Qualified DNA was used for library construction and sequencing.

Qualified genomic DNA was randomly sheared into approximately 350 bp fragments using a Covaris disruptor (Covaris Inc., Waltham, MA, USA). The fragments were end-repaired to generate 5′-phosphorylated blunt ends, 3′-A-tailed, and ligated to Illumina sequencing adapters using T4 DNA ligase. Two-step size selection was performed with the Agencourt SPRIselect kit, (Agilent Technologies (China) Co., Ltd., Shanghai, China.) to obtain a raw library with appropriate fragment lengths. After high-fidelity PCR amplification, library concentration was quantified with a Qubit 3.0 fluorometer, Thermo Fisher Scientific (China) Co., Ltd., Shanghai, China, insert size was checked using the Agilent 5400 system, and the effective concentration (1.5 nM) was accurately quantified by qPCR. Libraries that passed quality control were subjected to high-throughput sequencing on the Illumina NovaSeq 6000 platform with a PE150 (paired-end 150 bp) strategy after bridge PCR amplification on the chip, with a sequencing depth of ≥50×.

Raw sequencing reads were filtered by quality control to remove low-quality bases, N-bases and adapter-contaminated reads, yielding clean data. De novo genome assembly was performed using SOAP denovo (v2.04), SPAdes (v3.15.5) and Abyss (v2.3.5) with various K-mer values. Assembly results were integrated using CISA (v1.0), gap filling was conducted with gapclose, and low-coverage reads were filtered to obtain high-quality assembled sequences of ≥500 bp.

Using the *E. coli* ATCC 25922 reference genome as a control, whole-genome alignment was performed with MUMmer, and a collinearity map was drawn with Circos (v0.69-9) to analyze genomic structural variations. Protein-coding genes were predicted with GeneMarkS (v4.30). Interspersed and tandem repeats were identified using RepeatMasker (v4.1.5) and TRF (v4.09.1), respectively. Non-coding RNAs were annotated using tRNAscan-SE (v2.0.12), RNAmmer (v1.2) and Rfam (v1.2). Genomic islands, prophages and CRISPR sequences were predicted sequentially using IslandPath-DIOMB (v1.0), phiSpy (v2.3) and CRISPRdigger (v1.0).

The predicted coding genes were aligned against the GO, KEGG, COG and NR databases via BLASTp (v2.13.0) (e-value ≤ 1 × 10^−5^). Functional annotation was carried out using the hits with the highest score, identity ≥ 40% and coverage ≥ 40%. GO functional enrichment and KEGG pathway enrichment analyses were performed using GOATOOLS (v1.2.3) and KOBAS (v3.0), respectively. The annotated genes were also compared against the PHI and VFDB databases to analyze pathogen–host interaction genes and virulence factors. Antimicrobial resistance genes were annotated based on the CARD database to reveal the enrichment patterns of resistance genes and their correlations with phenotypes in strains induced by different disinfectants.
